# A rare concurrence of Muir-Torre-associated sebaceous carcinoma in the setting of a lipedematous scalp

**DOI:** 10.1080/23320885.2020.1833334

**Published:** 2020-10-19

**Authors:** Allison Shanks, Jake Laun, Amanda Holstein, Saksham Varshney, Jane Messina, Carl Wayne Cruse

**Affiliations:** a Department of Plastic Surgery, University of South Florida, Tampa, FL, USA; b Morsani College of Medicine, University of South Florida, Tampa, FL, USA; c Department of Cutaneous Oncology, H. Lee Moffitt Cancer Center and Research Institute, Tampa, FL, USA; d Department of Pathology, H. Lee Moffitt Cancer Center and Research Institute, Tampa, FL, USA

**Keywords:** Lipedematous scalp, Muir-Torre syndrome, sebaceous carcinoma

## Abstract

Muir-Torre syndrome (MTS) a genetic disorder characterized by predisposition to cutaneous neoplasms. Lipedematous scalp is characterized by the presentation of a thick, sponge-like scalp due to the altered and increased deposition of adipose tissue. We present a case of Muir-Torre-associated sebaceous carcinoma of the scalp consistent with a lipedematous scalp.

## Introduction

Sebaceous carcinomas are rare among the general population and are known to be characteristic of Muir Torre Syndrome (MTS). MTS is a genetic disorder that is characterized by a predisposition to sebaceous skin cancers, keratoacanthomas, as well as visceral cancers, most commonly gastrointestinal and genitourinary cancers; it is considered a variant of hereditary nonpolyposis colorectal cancer (HNPCC) or Lynch syndrome. Muir originally described MTS in 1967 with Torre reporting a patient in 1968 [1]. It was not until 1981 that Lynch recognized the connection with MTS and colorectal cancer [1]. This autosomal dominant condition is caused by mutations in one or more DNA mismatch repair (MMR) genes which predispose to microsatellite instability and genomic alterations leading to the development of the tumors. Diagnosis with MTS requires at least one sebaceous tumor and one visceral or internal organ cancer within the patient’s lifetime and not affected by other factors such as radiation or AIDS. Other important clinical factors that should be considered in the diagnosis of MTS are the following: age younger than 60 at presentation of first sebaceous tumor, total number of sebaceous neoplasms as well as a personal or family history of Lynch syndrome related cancers. Often, those with an unclear family history can be discovered through the presence of the skin lesion, similar to our patient [2,3]. The skin lesions may be benign or malignant tumors. Sebaceous carcinoma is historically categorized as tumors arising from ocular and extra-ocular origin (due to the high concentrations of sebaceous, Meibomian glands), with ocular accounting for 75 percent of all sebaceous carcinomas [4,5].

Originally described by Combleet in 1935, the term ‘lipedematous scalp’ was not introduced until Lee described it in 1994 in an attempt to separate it from lipedematous alopecia [6]. When a lipedematous scalp is accompanied by hair loss, it is described as lipedematous alopecia (LA), which includes painful and pruritic sections of the scalp that histologically appears with perivascular and periadnexal fibrosis and lymphocytic infiltrate [7]. LS and LA are most often found in African American adult women; however, there have been some reported cases of LA being found in younger patients as well as other races. The etiology as well as cause of the condition is unknown with only around 45 cases reported in the literature since the description by Lee in 1994 [8]. We present here a rare case report of a patient diagnosed with an MTS associated sebaceous carcinoma occurring in the setting of a lipedematous scalp.

## Case presentation

**Figure 1.**
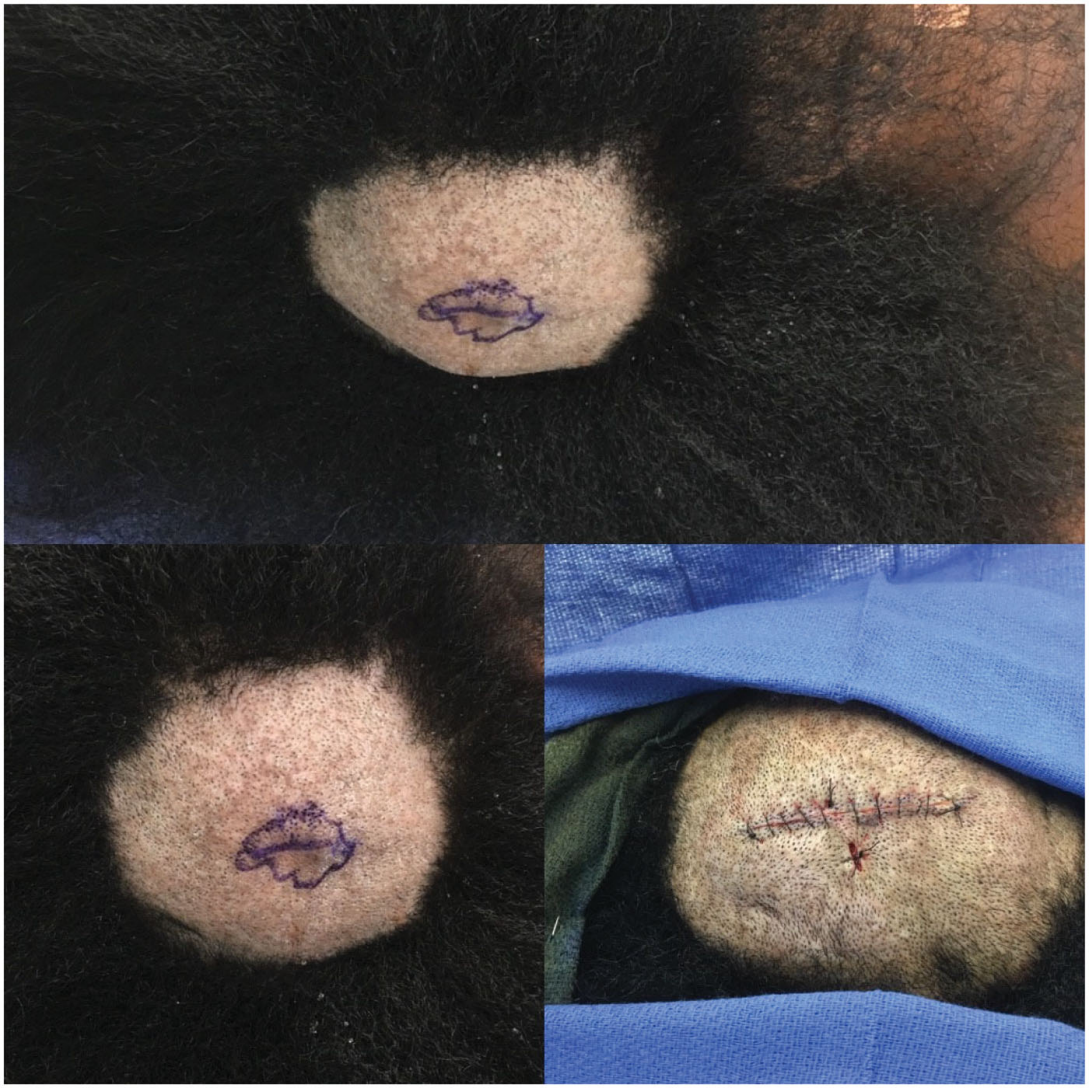
Top: Right posterior parietal scalp with skin lesion. Bottom left: Closer-up view of lesion and scalp. Bottom right: Post-excision as well as showing inferior biopsy site.

A healthy 37 year old African American female with no significant past medical history presented to the plastic surgery service for treatment of a recently diagnosed sebaceous carcinoma of the scalp. She denied any personal or family history of previous skin cancers or other malignancies. On first exam, the thickness of her scalp was not initially appreciated. However, during the surgical procedure, her scalp was noted to have a ‘sponge-like’ consistency and was noted to be thicker than normal tissue. An increased egress of fluid or increased bleeding was not noted during the operation. Due to the concern for possible satellite metastasis, a biopsy of her scalp was taken from the area inferior to the excision site (as seen in Figure 1) and submitted for frozen section, which was interpreted as negative for malignancy. The sebaceous carcinoma was then widely excised.

**Figure 2.**
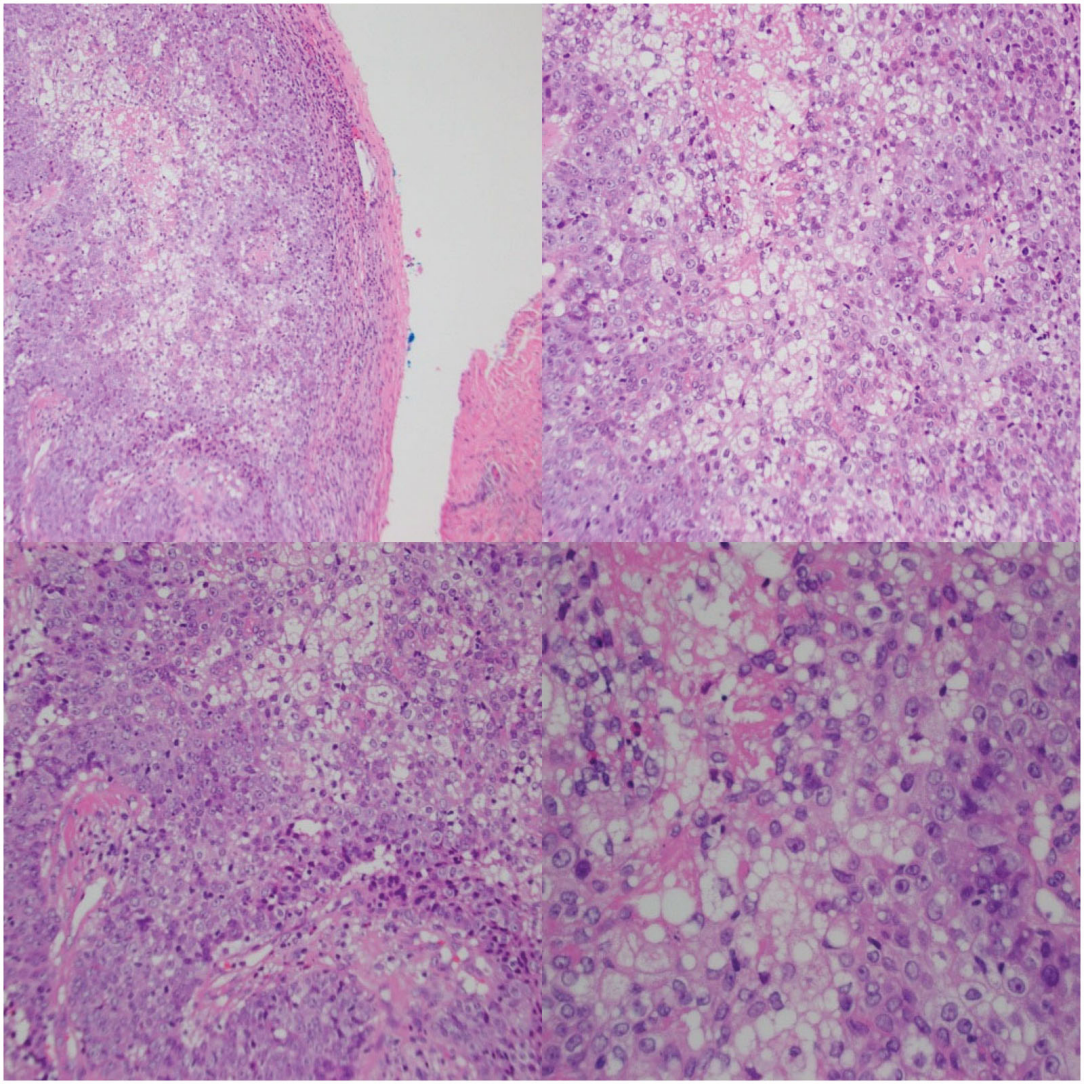
The tumor consists of lobules of basaloid to multivacuolated tumor cells, the latter containing clear vacuoles in the cytoplasm, indicating sebaceous differentiation. Top left: 100x magnification. Bottom left and Top right: 50x magnification. Bottom right: 200x magnification.

**Figure 3.**
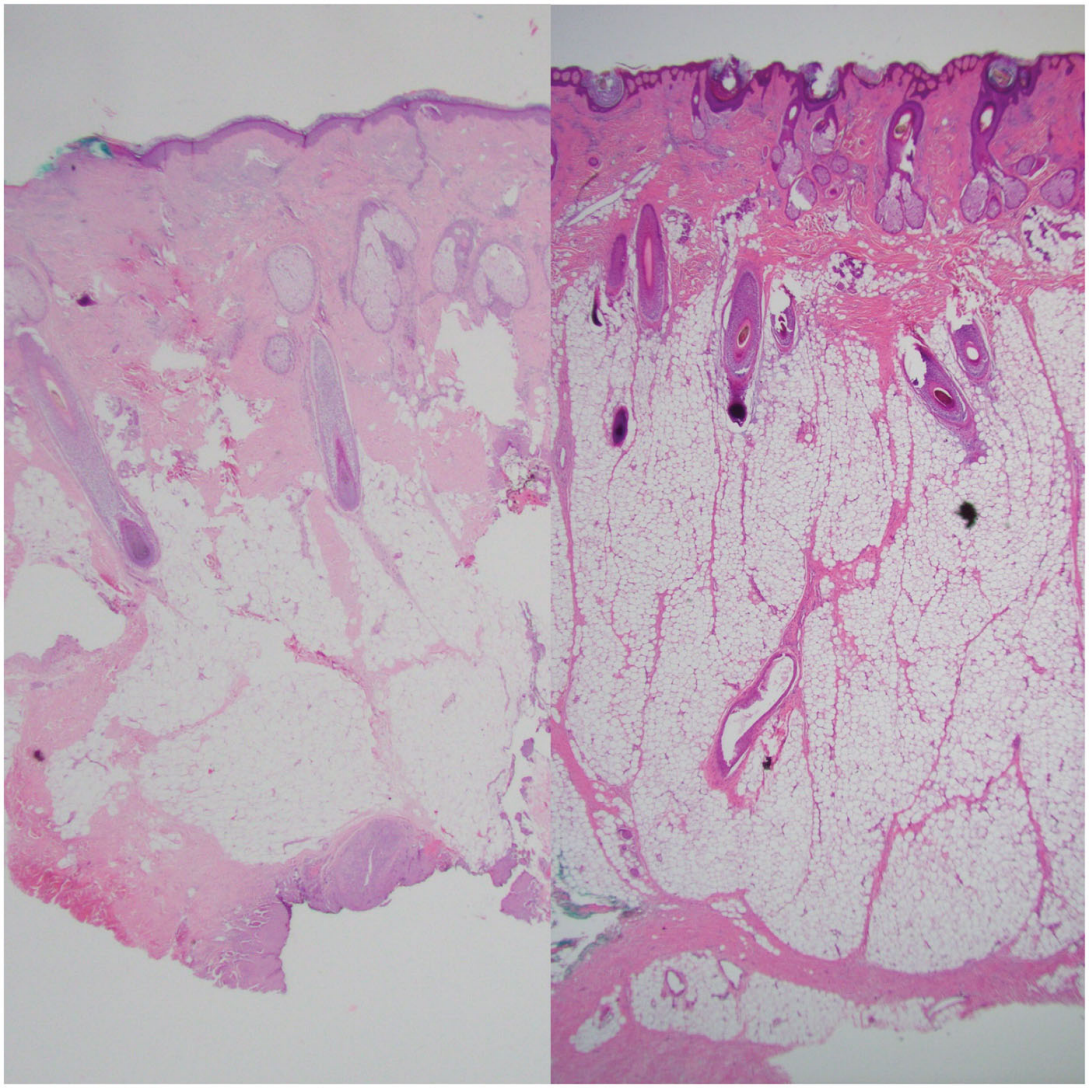
Left: pathology slide showing normal skin of the scalp. Right: pathology slide showing the thicker specimen, increased adipose tissue and edematous aspect of the lipedematous scalp.

Pathological assessment of the initial scalp biopsy and re-excision specimen revealed a sebaceous carcinoma characterized by positive staining for cytokeratin 5/6, p63, and bubbly positivity for epithelial membrane antigen. Representative histological slides can be seen in Figure 2. The tumor showed absence of staining for MSH-2 and MSH-6 with preserved staining for MLH-1 and PMS-2. In addition, the scalp biopsy and wide excision both showed an abnormal and diffuse thickening of the subcutaneous adipose layer with the patient’s scalp skin measuring 10.6 mm from epidermis to fascia, compared to a normal thickness of approximately 5–7 mm as can be seen in Figure 3. The adipocytes also appeared to encroach into the dermis, but were of normal size. There was an impression of mucin deposition between adipocytes; no significant inflammation was noted.

Because of the immunohistochemical loss of mismatch repair proteins, the patient was referred for genetic testing. This revealed a pathogenic variant in the MSH2 gene known a c.20delA, and the patient was diagnosed with Muir-Torre Syndrome. She subsequently underwent a colonoscopy that was found to be normal. Her post-operative course was uneventful and she did not have any issues post-operatively with wound healing and did not have any issues with local alopecia.

## Discussion

### Muir-Torre syndrome and sebaceous carcinomas

MTS is a rare genetic condition that predisposes to sebaceous skin conditions and visceral cancers, most frequently large bowel, endometrium, genitourinary tract, breast, and ovarian. The condition most commonly arises in an autosomal dominant inheritance pattern with a mutation in a DNA mismatch repair (MMR) gene – principally *MLH1 or MSH2* (found in 90% of MTS patients) that code for proteins that function to correct errors that occur during DNA replication [9]. These mutations ultimately lead to microsatellite instability and a predisposition for developing various malignancies [2,9]. Less commonly, around 35% of tumors arise in an autosomal recessive form as a result of a biallelic defect of the *MYH* gene which encodes base excision repair [10].

The most common sebaceous neoplasms in MTS are sebaceous adenomas, sebaceous epitheliomas, and sebaceous carcinomas with varying reports of frequency. Sebaceous adenomas as the initial presentation for MTS have reported associations from 25% to 60%. Sebaceous carcinomas in patients with MTS have reported frequencies of 36–100%, while sebaceous epithelioma associations range from 31% to 86%. Sebaceous neoplasms are rare among the general population and are the most defining characteristic of MTS. Specifically, sebaceous carcinoma represents less than 1% of all cutaneous malignancies and 17% of all sebaceous neoplasms in the general population [4,11]. Although reports vary, most studies state the average age at diagnosis occurs during the 6^th^ and 7^th^ decades of life, and women are more commonly affected than men, comprising about 70% of patients diagnosed with sebaceous carcinomas [12]. Twenty-three percent of sebaceous carcinomas are associated with MTS and MMR deficiency [11]. Sebaceous carcinoma associated with MTS has proven to be significantly more aggressive than tumors that occur spontaneously. A recent NCI SEER study of 3,299 cases of sebaceous carcinoma found that the 5-year cause-specific survival (mortality due to sebaceous carcinoma) was significantly decreased (from 78.2% to 52.5%) in patients with MTS compared to those with sebaceous carcinoma exclusively [13]. Extraocular sebaceous carcinomas account for one-fourth of all sebaceous carcinomas with about 70% found on the head and scalp due to high concentration of sebaceous glands [4,5,12].

Clinical presentations of sebaceous carcinomas are varied, and it is known for masquerading as common benign conditions. In a study of 31 patients with extraocular sebaceous carcinoma, the most common presentations were blepharoconjunctivitis (32%), eyelid masses (29%), or eyelid thickening (26%), with regional lymphadenopathy accounting for 3% of initial presentations [14]. Extraocular sebaceous carcinomas usually present similarly to benign skin conditions like pyoderma gangrenosum or molluscum contagiosium as well as to malignant skin lesions like squamous cell carcinoma or basal cell carcinoma [15]. Due to the variety of clinical presentations, sebaceous carcinoma is frequently misdiagnosed. These misdiagnoses lead to delay in treatment and increased risk of local recurrence, metastasis, and poor outcomes.

Sebaceous carcinomas have the ability to invade and produce metastases; therefore, it is often recommended to excise with 5–6 mm margins. Lymph node investigation is sometimes recommended for larger and more invasive tumors due to the 3–28% risk for local metastasis [2,15]. Colonoscopies are also recommended as early as 18–20 years old due to increased risk of GI malignancies [2].

### Lipedematous scalp

Another condition seen in our patient was the presence of a lipedematous scalp (LS). LS as well as lipedematous alopecia (LA) are rare conditions that are, as stated previously, most often seen in African-American women. Due to the increased proportion of women affected, hormonal factors have been hypothesized to have some level of influence [8]. Theories have been proposed as to the role of leptin in adipose distribution and development; however, nothing has been proven as of yet [6]. LS and LA have been associated with some other disease processes such as discoid lupus, diabetes mellitus, as well as hyperelastic skin conditions [8].

Lipedematous scalp is characterized by a thickened subcutaneous and adipose layer in the scalp that presents clinically as a soft, almost sponge-like consistency to the scalp with thickened areas mainly in the scalp vertex and occiput. The lipedematous scalp is measurably thicker (∼10–16mm) than the normal scalp (∼5–6mm) as can be seen in our biopsy specimens above in Figure 3 [8]. Treatment is also debatable with no specific guidelines being proposed. Intra-lesional steroids as well as surgical debulking have been proposed with some success [6,16]. One of the main differential diagnoses is cutis verticis gyrate (CVG) which can be separated from LS and LA in that CVG mainly affects men and is characterized by a thick scalp and shows thickened collagen in the dermis, unlike LS and LA which shows an unaffected dermis and normal epidermis [16].

## Conclusion

Sebaceous carcinomas are rare and, when present, should encourage the investigation into possible connections with syndromes such as Muir-Torre Syndrome. MTS is associated with an increased risk for other malignancies, especially GI/GU, and warrants a thorough investigation to rule out the presence of any other tumors. A lipedematous scalp is a very rare finding that has only recently been reported and does not appear to have any connection with sebaceous carcinoma or MTS. We therefore present this unique case that has two rare skin related conditions, sebaceous carcinoma associated with MTS and a lipedematous scalp.

## Learning points

- Sebaceous carcinomas are associated with patients that have Miur-Torre syndrome.
- Muir-Torre syndrome places patients at risk for malignancy, especially GI/GU malignancies and they require more surveillance than the general population.
- A lipedematous scalp may be one way of presentation of sebaceous carcinoma, especially in regards to Muir-Torre patients.

## References

[1] Ponti G, Ponz de Leon M. Muir-Torre syndrome. Lancet Oncol. 2005;6:980–987. 16321766 10.1016/S1470-2045(05)70465-4

[2] John AM, Schwartz RA. Muir-Torre syndrome (MTS): an update and approach to diagnosis and management. J Am Acad Dermatol. 2016; 74:558–566. 26892655 10.1016/j.jaad.2015.09.074

[3] Roberts ME, Riegert-Johnson DL, Thomas BC, et al. A clinical scoring system to identify patients with sebaceous neoplasms at risk for the Muir-Torre variant of Lynch syndrome. Genet Med. 2014;16:711–716. 24603434 10.1038/gim.2014.19

[4] Ghosh SK, Gupta D, Chatterjee S, et al. Rapidly growing extraocular sebaceous carcinoma occurring during pregnancy: a case report. Dermatol Online. 2008;14:8. 19061568

[5] Bhavarajua VMK, Shamim SE, Naik VR, et al. Sebaceous cell carcinoma of scalp – a rare presentation. MJMS. 2007;14:67–70. PMC3351222 22593656

[6] Al Gaadi S, Al Godayan S, Bukhari I. Lipedematous scalp: case report and review of the literature. Our Dermatol Online. 2016;7:198–200.

[7] Cabrera R, Larrondo J, Whittle C, et al. Successful treatment of lipedematous alopecia using mycophenolate mofetil. Acta Derm Venereol. 2015;95:1011–1012. 25881615 10.2340/00015555-2114

[8] Lee HE, Kim SJ, Im M, et al. Congenital lipedematous alopecia: adding to the differential diagnosis of congenital alopecia. Ann Dermatol. 2015;27:87–89. 25673939 10.5021/ad.2015.27.1.87 PMC4323610

[9] Machin P, Catasus L, Pons C, et al. Microsatellite instability and immunostaining for MSH-2 and MLH-1 in cutaneous and internal tumors from patients with the Muir-Torre syndrome. J Cutan Pathol. 2002;29:415–420. 12139636 10.1034/j.1600-0560.2002.290705.x

[10] Perera S, Ramyar L, Mitri A, et al. A novel complex mutation in MSH2 contributes to both Muir-Torre and Lynch Syndrome. J Hum Genet. 2010;55:37–41. 19911012 10.1038/jhg.2009.119

[11] Walsh MD, Jayasekara H, Huang A, et al. Clinico-pathological predictors of mismatch repair deficiency in sebaceous neoplasia: a large case series from a single Australian private pathology service. Australas J Dermatol. 2019;60:126–133. 30506759 10.1111/ajd.12958

[12] Shields JA, Demirci H, Marr BP, et al. Sebaceous carcinoma of the ocular region: a review. Surv Ophthalmol. 2005;50:103–122. 15749305 10.1016/j.survophthal.2004.12.008

[13] Tripathi R, Bordeaux JS. Impact of Muir-Torre Syndrome on survival in patients with sebaceous carcinoma: a SEER population-based study. Dermatol Surg. 2019;45:148–149. 29578884 10.1097/DSS.0000000000001503

[14] Song A, Carter KD, Syed NA, et al. Sebaceous cell carcinoma of the ocular adnexa: clinical presentations, histopathology, and outcomes. Ophthalmic Plast Reconstr Surg. 2008;24:194–200. 18520834 10.1097/IOP.0b013e31816d925f

[15] Knackstedt T, Samie FH. Sebaceous carcinoma: a review of the scientific literature. Curr Treat Options Oncol. 2017;18:47. 28681210 10.1007/s11864-017-0490-0

[16] Carrasco-Zuber JE, Alvarez-Veliz S, Cataldo-Cerda K, et al. Lipedematous scalp: a case report and review of the current literature. J Dtsch Dermatol Ges. 2016;14:418–421. 27018637 10.1111/ddg.12813

